# Dormancy-like Phenotype of *Aggregatibacter actinomycetemcomitans*: Survival during Famine

**DOI:** 10.3390/pathogens13050418

**Published:** 2024-05-16

**Authors:** Natalia O. Tjokro, Carolyn B. Marks, Ashley Wu, Casey Chen

**Affiliations:** 1 Department of Endodontics and Periodontics, Herman Ostrow School of Dentistry, University of Southern California, 925 W. 34th Street, Los Angeles, CA 90089, USA; ntjokro@ostrow.usc.edu (N.O.T.); ashley99wu@gmail.com (A.W.); 2 Core Center of Excellence in Nano Imaging, University of Southern California, 1002 Childs Way, Los Angeles, CA 90089, USA; markscar@usc.edu

**Keywords:** *Aggregatibacter actinomycetemcomitans*, dormancy, stress response, bacterial physiology

## Abstract

Microbes frequently experience nutrient deprivations in the natural environment and may enter dormancy. *Aggregatibacter actinomycetemcomitans* is known to establish long-term infections in humans. This study examined the dormancy-like phenotype of an *A. actinomycetemcomitans* strain D7S-1 and its isogenic smooth-colony mutant D7SS. A tissue culture medium RPMI-1640 was nutrient-deficient (ND) and unable to support *A. actinomycetemcomitans* growth. RPMI-1640 amended with bases was nutrient-limited (NL) and supported limited growth of *A. actinomycetemcomitans* less than the nutrient-enriched (NE) laboratory medium did. Strain D7S-1, after an initial 2-log reduction in viability, maintained viability from day 4 to day 15 in the NL medium. Strain D7SS, after 1-log reduction in viability, maintained viability from day 3 to day 5. In contrast, bacteria in the NE medium were either non-recoverable (D7S-1; >6-log reduction) or continued to lose viability (D7SS; 3-log reduction) on day 5 and beyond. Scanning and transmission electron microscopy showed that *A. actinomycetemcomitans* in the NL medium formed robust biofilms similar to those in the NE medium but with evidence of stress. *A. actinomycetemcomitans* in the ND medium revealed scant biofilms and extensive cellular damage. We concluded that *A. actinomycetemcomitans* grown in the NL medium exhibited a dormancy-like phenotype characterized by minimum growth, prolonged viability, and distinct cellular morphology.

## 1. Introduction

Most studies on microbial physiology and metabolism have been performed under conditions of nutrient excess. However, microbes are frequently starved for critical growth nutrients in most natural environments [1]. Bacteria may enter a dormancy state in unfavorable environments to ensure long-term survival. Dormancy is a reversible phase in which bacteria are viable but non-replicating and exhibit low levels of metabolic activity with distinct gene expression profiles [1].

*Aggregatibacter actinomycetemcomitans* is among the best-studied periodontal pathogens. The organism is noted for its association with periodontitis in young individuals and adults with severe periodontitis [2,3]. *A. actinomycetemcomitans* can establish long-term periodontal infections due to persistent subgingival colonization of identical *A. actinomycetemcomitans* clones [4,5]. The mechanism that underpins the organism’s persistent colonization remains to be elucidated. Presumably, *A. actinomycetemcomitans* may enter a dormancy phase in vivo when it encounters unfavorable conditions.

We hypothesize that *A. actinomycetemcomitans* may enter dormancy in the in vivo nutrient-limited environment. The in vivo milieu of *A. actinomycetemcomitans* is complex. It involves bacteria- and host-derived factors such as the polymicrobial community and its metabolites, gingival crevicular fluid, various immune cells, cytokines, and chemokines [6,7,8,9]. Developing an in vitro model that mimics the in vivo environment is challenging and nearly impossible. Instead, this study examined the potential dormancy phenotype of *A. actinomycetemcomitans* in a relatively simple model by growing bacteria in chemically defined media (CDMs) and characterizing its phenotype. *A. actinomycetemcomitans* in dormancy, in comparison to *A. actinomycetemcomitans* grown in the conventional nutrient-enriched medium, is expected to (i) display no or minimum growth, (ii) remain viable over an extended time, and (iii) demonstrate a distinct cellular morphology. The phenotypes and the mechanism of dormancy will be further tested in animal models in future investigations.

## 2. Materials and Methods

### 2.1. Bacterial Strains and Growth Conditions

Bacteria were routinely incubated in an atmosphere supplemented with 5% CO_2_ at 37 °C in a humidified incubator. The wildtype rough-colony *A. actinomycetemcomitans* strain D7S-1 and its isogenic smooth-colony mutant D7SS were routinely grown in a modified trypticase soy broth (mTSB) containing 3% trypticase soy broth and 0.6% yeast extract or on mTSB agar (mTSB with 1.5% agar (Becton Dickinson and Company, Franklin Lakes, NJ, USA)).

Three CDMs were tested in the study. A common tissue culture medium, RPMI-1640 (Sigma, St. Louis, MO, USA, Catalog #: R0883), designated as RPMI-1, was used as a CDM base. RPMI-2 was RPMI-1 amended with 10 mg/L uracil and 10 mg/L hypoxanthine. RPMI-3 was RPMI-2 amended with 0.03 mM each of cytidine, guanosine, uridine, adenosine, and thymidine (Sigma, ES-008-D), 10 mM fructose, 2 g/L inosine, 2 mg/L spermidine, 2 mg/L putrescine, 430 mg/L MgCl_2_, and 2.2 mg/L CaCl_2_.

Three different protocols were used to prepare *A. actinomycetemcomitans* for electron microscopy: (i) biofilms grown on glass coverslips for SEM, (ii) a bacterial suspension from liquid cultures for SEM, and (iii) bacteria pelleted from liquid cultures for TEM. Briefly, colonies of *A. actinomycetemcomitans* on agar were inoculated into mTSB broth and incubated overnight. Cells were washed with sterile PBS buffer and adjusted to OD_600_ of 0.15 in three test media (i.e., mTSB control, RPMI-1, and RPMI-2). Sterile coverslips were then added to the cultures to support biofilm formation. Cells were collected and prepared for SEM or TEM at designated times.

### 2.2. Electron Microscopy

Biofilms on coverslips for SEM and the pelleted cells for TEM were fixed by replacing the media with a solution containing 2.5% glutaraldehyde, 2% paraformaldehyde, and 2% sucrose (*w*/*v*) in 0.1 M HEPES. The bacterial suspension from cultures was fixed for SEM by adding 100 µL of 25% glutaraldehyde directly to 900 µL of the culture for a final concentration of 2.5% glutaraldehyde. All samples were processed in a Pelco BioWave microwave processor (Ted Pella, Reading, CA, USA) to increase the penetration of samples and decrease processing time, using a Cool Spot to control temperature and reduce standing energy waves. All samples were microwaved for two cycles of 1 min on, 1 min off, and 1 min on at 150 watts, and then this sequence was repeated at 250 w. The samples were stored at 4 °C.

### 2.3. TEM

Pellets were microwaved with wash buffer (0.1 M HEPES containing 2% sucrose) for 1 min at 250 w. This was repeated three times before quenching with fresh 50 mM ammonium chloride in wash buffer for two cycles of 1 min on, 1 min off, and 1 min at 150 w under vacuum. This was followed by two wash rinses for one minute each at 250 w, then two 18 mΩ water rinses for one minute each at 250 w. Pellets were stained with 1% uranyl acetate in water under vacuum (2 min on, 2 min off, and 2 min on; two cycles at 100 w), followed by two 18 mΩ water rinses before a graded alcohol dehydration series (50%, 70%, 95%, 100%, 100%, and 100%) at 250 w for 1 min at each step.

Before infiltrating the samples with Embed 812 (EMS) epoxy resin, samples were rinsed twice with propylene oxide. BioWave was used for the first two resin dilutions (1:1 and 1:3 of propylene oxide to Embed 812) for 4 min at 250 w. Three pure resin changes followed this before placing the samples into flat molds with paper labels. Blocks were polymerized at 60 °C. Eighty-nanometer sections were cut using a Leica UC6 ultramicrotome (Leica, Wetzlar, Germany) and a Diatome diamond knife. Sections were picked up on 200-mesh thin bar Gilder grids (EMS).

All TEM images were taken using a ThermoFisher (Waltham, MA, USA) Talos FEG TEM at 80 KeV and recorded on a ThermoFisher Ceta16 CMOS camera.

### 2.4. SEM

Biofilms grown on coverslips were processed the same way as the TEM pellets up to the final dehydration step. After dehydration, the cells adherent to the cover glasses were dried by replacing the 100% ethanol with 100% hexamethyldisilazane (HMDS) three times. With each HMDS change, the samples were microwaved for 45 s at 150 w. Excess HMDS was removed, and samples were dried completely at 37 °C.

The cells in suspension were plated onto poly-l-lysine-coated 12 mm round cover glasses. After a 20-min incubation, samples were processed in terms of the cells grown on cover glass, with the following changes: no Quench step, no UA staining, and Critical Point Dried (Auto-Samdri, Tousimis, Rockland, MD, USA). All SEM samples were mounted to stubs using double-sided carbon sticky tabs and then sputter-coated for 40 s with an 80/20 platinum/palladium target (Cressington 108C Sputter coater, Ted Pella, Reading, CA, USA).

All SEM images were taken on an FEI Nova Nano 50 SEM (Hillsboro, OR, USA) using an Everhart–Thornley detector or a through-the-lens secondary electron detector in immersion mode.

## 3. Results

### 3.1. Development of Culture Media That Induce A. actinomycetemcomitans Dormancy-like Phenotype

We hypothesized that specific media may induce an *A. actinomycetemcomitans* dormancy-like phenotype characterized by minimal replication and sustained viability. A common tissue culture medium RPMI-1640, henceforth RPMI-1, was selected as the base of CDMs and further amended to derive RPMI-2 and RPMI-3. We first tested whether the CDMs were nutrient-limited (NL) or nutrient-deficient (ND) for *A. actinomycetemcomitans*. The NL media should possess all essential factors and can sustain the growth of bacteria indefinitely if fresh media are added periodically. In contrast, the ND media lack crucial elements and will not support the growth of bacteria with or without replenishment with fresh media. A modified trypticase soy broth with yeast extract medium (mTSB) was used as the control nutrient-enriched (NE) medium. Both *A. actinomycetemcomitans* D7S-1 and D7SS strains were used in our study to examine their phenotypic differences in different culture media. The comparison between the wildtype biofilm-forming bacteria and the smooth-colony mutant of planktonic bacteria is not the focus of this study.

**Figure 1:**
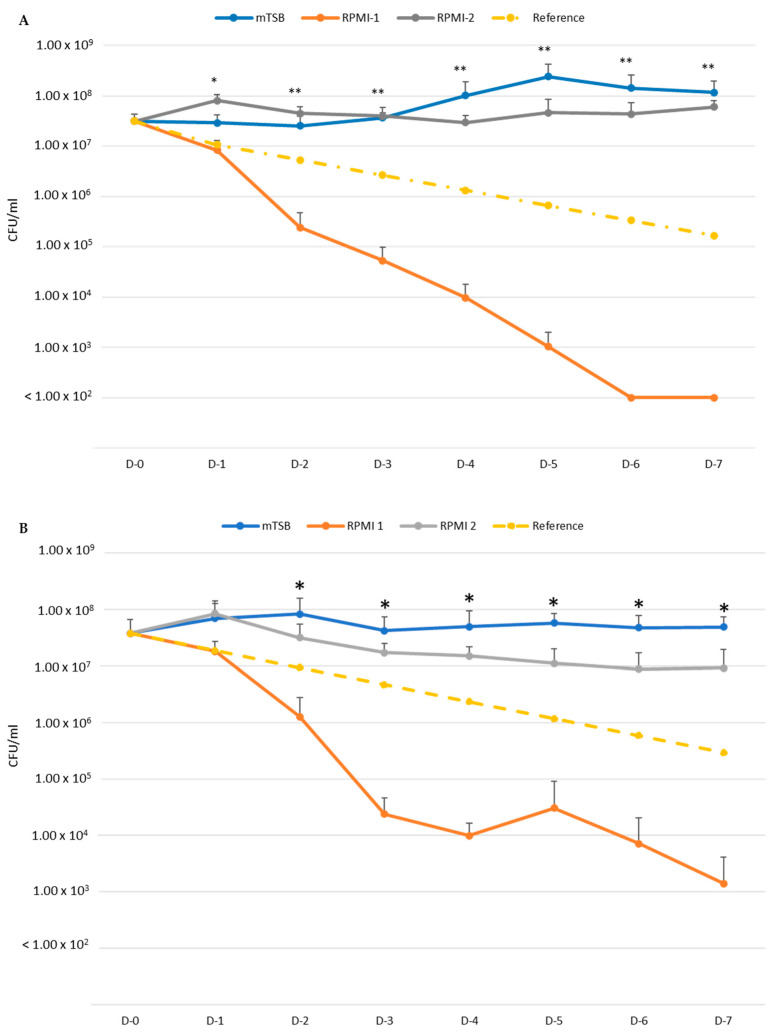
Distinguishing nutrient-deficient and nutrient-limited properties of CDMs. The CFU/mL was determined for *A. actinomycetemcomitans* D7S-1 (**A**) and *A. actinomycetemcomitans* D7SS (**B**) in mTSB, RPMI-1, and RPMI-2, with daily 1:1 dilution with appropriate fresh media. For *A. actinomycetemcomitans* D7S-1: * D-1: ANOVA 0.005419; Tukey HSD Test: RPMI-1 vs. RPMI-2, *p* < 0.01. ** D-2, D-3, D-4, D-5, D-6, and D-7: ANOVA < 0.005; Tukey HSD Test: mTSB vs. RPMI-1, *p* < 0.01; RPMI-1 vs. RPMI-2, *p* < 0.01. For *A. actinomycetemcomitans* D7SS: * D-2, D-3, D-4, D-5, D-6, and D-7: ANOVA < 0.002; Tukey HSD Test: mTSB vs. RPMI-1, *p* < 0.01: RPMI-1 vs. RPMI-2 *p* < 0.01.

Figure 1 shows the results of the experiments for strain D7S-1 (A) and D7SS (B) in mTSB, RPMI-1, and RPMI-2. The cultures were diluted 1:1 daily with fresh media for seven days. The dashed reference line represents the CFU/mL if the cultures did not grow or lose viability. Because of the daily dilution of the cultures with fresh media, the CFU of *A. actinomycetemcomitans* is expected to be reduced by 50% every day without gains or losses of cultivable bacteria. Three patterns of growth were observed. As expected, *A. actinomycetemcomitans* grew best in mTSB and was the lowest in RPMI-1. *A. actinomycetemcomitans* in RPMI-2 exhibited less growth than in mTSB. In particular, D7SS in RPMI-2 grew closer to the reference line of no gains and losses. The results suggest that RPMI-1 is an ND medium, and RPMI-2 is an NL medium. The growth of *A. actinomycetemcomitans* in RPMI-3 (RPMI-2 amended with dNTPs) was better than in RPMI-2 but worse than in mTSB (data not shown) and was not further tested.

**Figure 2:**
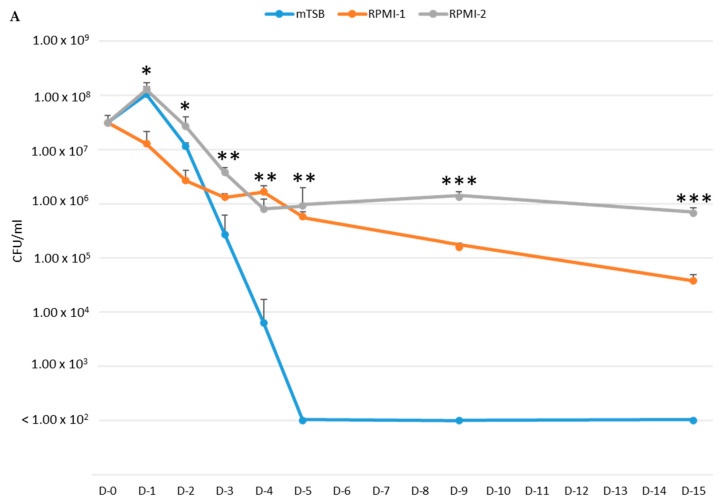

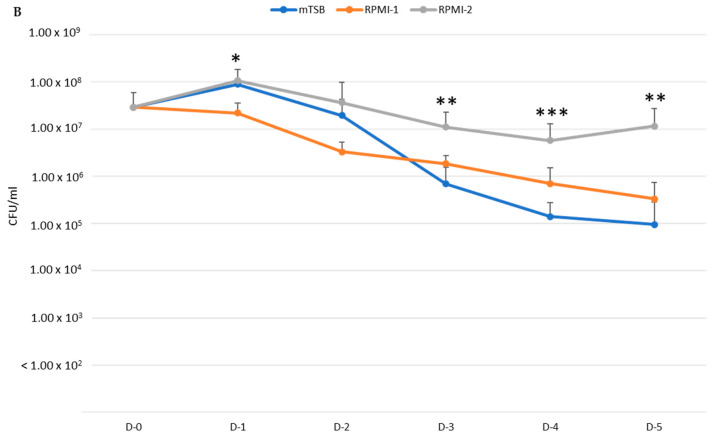
Dormancy-like phenotype of *A. actinomycetemcomitans* in nutrient-limited media. The viability of *A. actinomycetemcomitans* D7S-1 (**A**) and *A. actinomycetemcomitans* D7SS (**B**) in mTSB, RPMI-1, and RPMI-2 without daily addition of fresh media was determined by culturing. For *A. actinomycetemcomitans* D7S-1: * D-1 and D-2: ANOVA < 0.005; Tukey HSD Test: mTSB vs. RPMI-1, *p* < 0.01; RPMI-1 vs. RPMI-2, *p* < 0.01. ** D-3, D-4, and D-5: ANOVA < 0.02; Tukey HSD Test: mTSB vs. RPMI-1, *p* < 0.05; mTSB vs. RPMI-2, *p* < 0.01. *** D-9 and D-15: ANOVA < 0.0001; Tukey HSD Test: mTSB vs. RPMI-1, *p* < 0.01; mTSB vs. RPMI-2, *p* < 0.01; RPMI-1 vs. RPMI-2, *p* < 0.01. For *A. actinomycetemcomitans* D7SS: * D-1: ANOVA *p* = 0.034030; Tukey HSD Test: RPMI-1 vs. RPMI-2, *p* < 0.05. ** D-3 and D-5: ANOVA *p* < 0.002; Tukey HSD Test: mTSB vs. RPMI-2, *p* < 0.01. *** D-4: ANOVA *p =* 0.000772; Tukey HSD Test: mTSB vs. RPMI-2, *p* < 0.01; RPMI-1 vs. RPMI-2, *p* < 0.05.

We next tested if *A. actinomycetemcomitans* may reflect a dormancy-like phenotype in mTSB, RPMI-1, and RPMI-2 without adding fresh media. Three patterns of growth were also observed (Figure 2). *A. actinomycetemcomitans* D7S-1 cultured in mTSB, following an initial growth, was reduced by more than 2 logs on day 3 and was not cultivable (<100 CFU/mL; >6 logs of reduction) from day 5 to day 15. *A. actinomycetemcomitans* in RPMI-1 showed a continuing decline in viability from day 0 to day 15, when it exhibited about an 800-fold reduction in CFU/mL. Interestingly, *A. actinomycetemcomitans* in RPMI-2, following an initial growth on day 1, showed a decline in viability from day 1 to day 4 (less pronounced than in mTSB) and a period of stable viability from day 5 to day 15 that resembled dormancy. Moreover, the viability of *A. actinomycetemcomitans* in RPMI-2 exceeded 6000-fold greater than in mTSB on day 15. *A. actinomycetemcomitans* D7SS was tested in the three media for five days and showed similar growth trends to those observed for D7S-1. The bacteria exhibited initial growth on day 1 and a rapid decline in viability from day 2 to day 5 in mTSB, and a slower but continuing decrease in viability in RPMI-1 from day 0 to day 5. In contrast, bacteria in RPMI-2 showed initial growth, followed by a slow decline in viability from day 1 to day 3, when it reached a stable period of viability from day 3 to day 5, resembling dormancy. The viability of D7SS in RPMI-2 was 100-fold higher than in mTSB on day 5. The results suggested that *A. actinomycetemcomitans* entered a dormancy-like phase 3 to 4 days after culturing in RPMI-2 and demonstrated sustained viability for up to 15 days.

### 3.2. Scanning Electron Microscopy (SEM) of A. actinomycetemcomitans

We next examined the cellular morphology of *A. actinomycetemcomitans* cultured in mTSB, RPMI-1, and RPMI-2. The protocol and the list of samples are summarized in Supplementary Figure S1 and Table 1.

**Figure 3:**
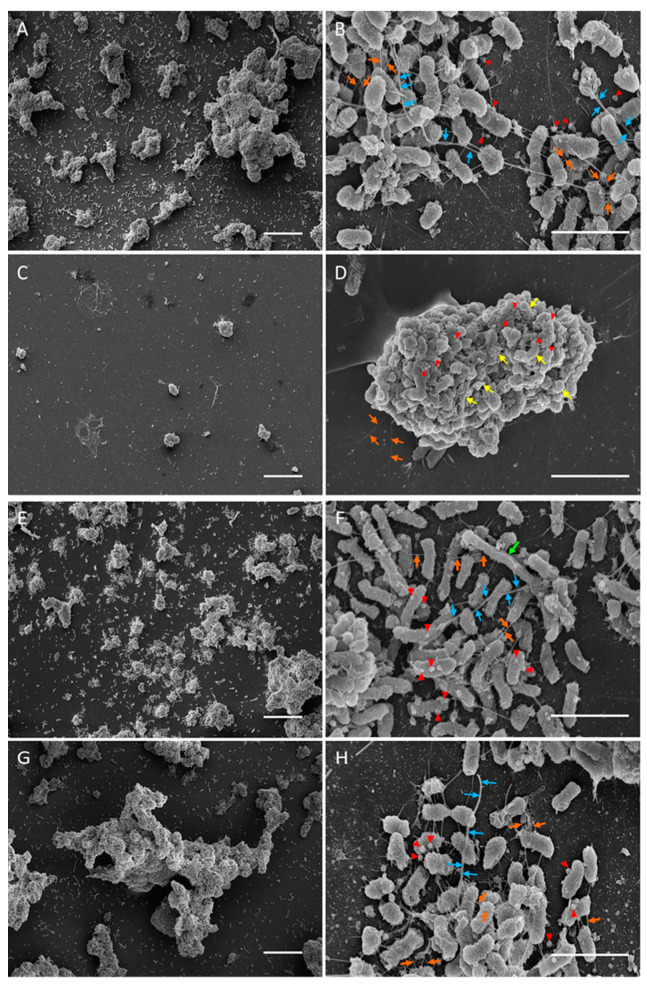
SEM of D7S-1 biofilms formed on the coverslips. The bacteria were cultured with a daily dilution of fresh media for four days in the mTSB control (**A**,**B**), RPMI-1 (**C**,**D**), RPMI-2 (**E**,**F**), or switched from RPMI-2 on day 4 to mTSB and grown with a daily dilution with fresh media until day 7 (**G**,**H**). Orange arrows indicate thin fibrils of the fimbriae, while blue arrows indicate fimbriae in thick bundles. Vesicles are represented by red triangles, and long cells are represented by green arrows. Yellow arrows indicate broken or irregularly shaped cells. The scale bars on the left and right columns are 20 and 2 µm, respectively.

In mTSB, D7S-1 grew as aggregates of bacteria of 1–2 µm in length (Figure 3A,B). The cells were intact. Numerous fimbria appeared as thin fibrils (orange arrows) or thick bundles (blue arrows) comprised of multiple strands of fimbriae. Moderate amounts of vesicles (~0.1 µm) (red triangles) were noted. In RPMI-1, D7S-1 showed fewer and smaller cell aggregates on the coverslips and fewer fimbriae (Figure 3C,D). Numerous cells appeared either broken or irregularly shaped (yellow arrows). More vesicles of varying sizes were noted. D7S-1 grew less in RPMI-2 than in mTSB but more than in RPMI-1 (Figure 3E,F). The cells also formed aggregates attached to the coverslip, similar to those in mTSB. The cells were intact and demonstrated numerous vesicles of irregular shapes. Both thin and thick fimbriae were noted. A few long cells (3–4 µm) (green arrows) were also found. The cells reverted to the morphology observed in the mTSB control after three days of culturing with the daily dilution of fresh media (Figure 3G,H).

**Figure 4:**
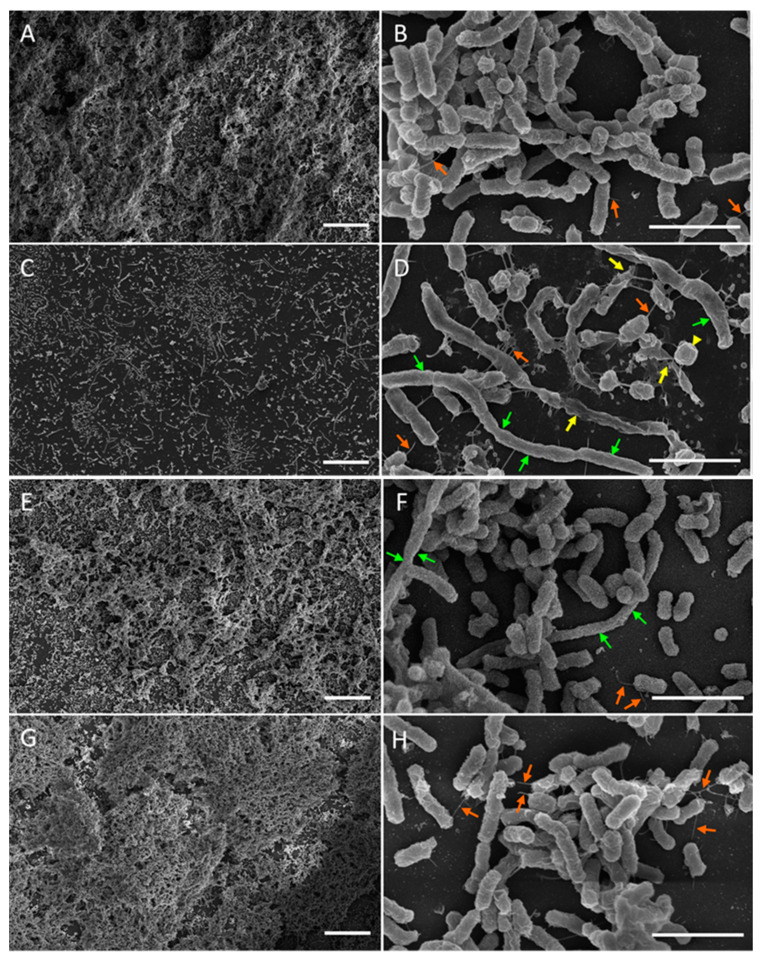
SEM of D7SS. The bacteria were cultured with a daily dilution of fresh media for four days in the mTSB control (**A**,**B**), RPMI-1 (**C**,**D**), RPMI-2 (**E**,**F**), or switched from RPMI-2 on day 4 to mTSB and grown with a daily dilution of fresh media until day 7 (**G**,**H**). Orange arrows represent thin fimbriae, and yellow arrows indicate broken or irregularly shaped cells. Long cells are denoted by green arrows, while yellow triangles indicate short and round cells. The scale bars on the left and right columns are 20 and 2 µm, respectively.

The morphology of the isogenic smooth-colony mutant D7SS differed from that of D7S-1 in all media tested (Figure 4). In mTSB, D7SS grew as a thick mat of cells. The cells were longer (up to 4 µm) compared to D7S-1 (Figure 4A,B). The cells were intact, with a few vesicles. A few thin fimbriae (orange arrows) (but not the thick bundles of fimbriae) were noted. D7SS in RPMI-1 grew less than in mTSB and exhibited numerous extra-long cells (green arrows) (~10 µm), which may comprise undivided chains of cells (Figure 4C,D). Some of the long cells showed evidence of damage (yellow arrows). A few thin fimbriae (orange arrows) were noted. There were also numerous short and round cells (yellow triangles) not seen in other samples.

D7SS in RPMI-2 grew as well as in mTSB and was much better than in RPMI-1 (Figure 4E,F). Extra-long cells (green arrows) were found but less numerous than in RPMI-1. Notably, the cells appeared to be largely intact. The small round cells found in RPMI-1 cultures were not present in RPMI-2, and thin fimbriae (orange arrows) were occasionally seen. The morphology of D7SS in RPMI-2 upon culturing in mTSB for three days was indistinguishable from that in mTSB only (Figure 4G,H).

### 3.3. Transmission Electron Microscopy (TEM) of A. actinomycetemcomitans

**Figure 5:**
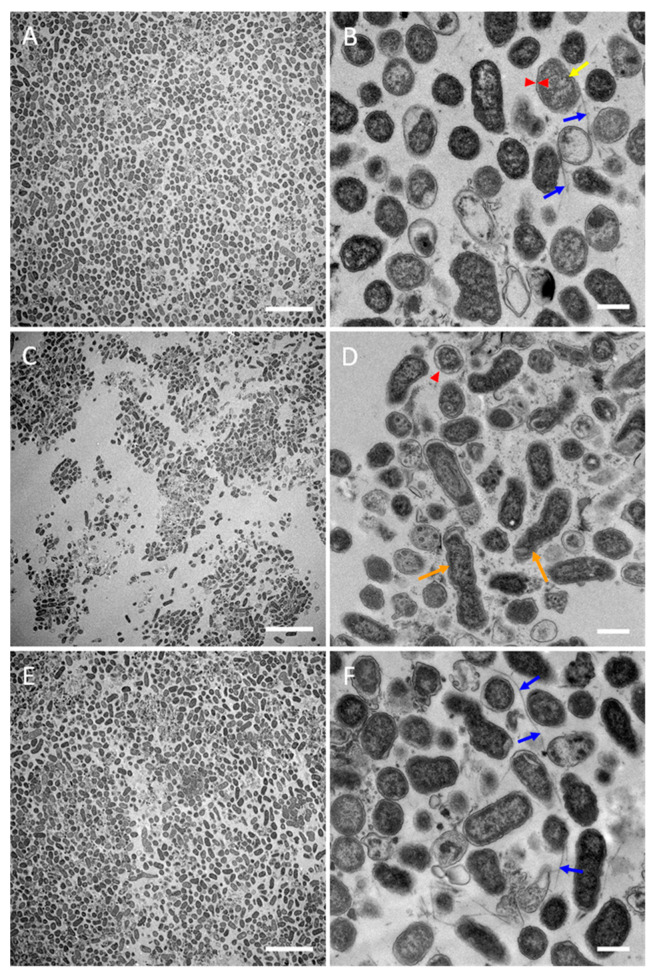
TEM of D7S-1. The bacterial growth conditions are described in Figure 3. The bacteria were pelleted from the cultures and processed for TEM. Intact D7S-1 cells in RPMI-1 could not be found based on TEM, likely due to the low number of cells in the cultures. The samples included D7S-1 in the mTSB control (panels **A**,**B**) and in RPMI-2 (panels **C**,**D**), and the bacteria switched from RPMI-2 to mTSB (panels **E**,**F**). Cellular cytoplasmic membranes, periplasmic space, and outer membranes are indicated by red triangles, while yellow arrows highlight the pale-staining area occupied by genomic DNA. Blue arrows denote thick fimbriae, and extra-long cells are indicated by orange arrows. The scale bars on the left and right columns are 5 µm and 500 nm, respectively.

The TEM of D7S-1 in the mTSB control showed intact cells (Figure 5A,B) with well-defined cytoplasmic membranes, periplasmic space, and outer membranes (red triangles). The cytoplasm was characterized by a pale-staining area of DNA (yellow arrows) and numerous ribosomes. Thick fimbriae (blue arrows) were also found. We could not find intact D7S-1 in RPMI-1 based on TEM, likely due to the low number of cells in the cultures. D7S-1 in RPMI-2 showed fewer cells than in the mTSB control (Figure 5C,D). However, the cells were largely intact, with well-defined cytoplasmic membranes, periplasmic space, and outer membranes (red triangles). In general, the cytoplasm contained fewer dark-staining ribosomes than in the mTSB control. A few extra-long cells (orange arrows) were noted. D7S-1 in RPMI-2, when cultured in mTSB, reverted to the morphology noted in cells cultured in mTSB only (Figure 5E,F).

**Figure 6:**
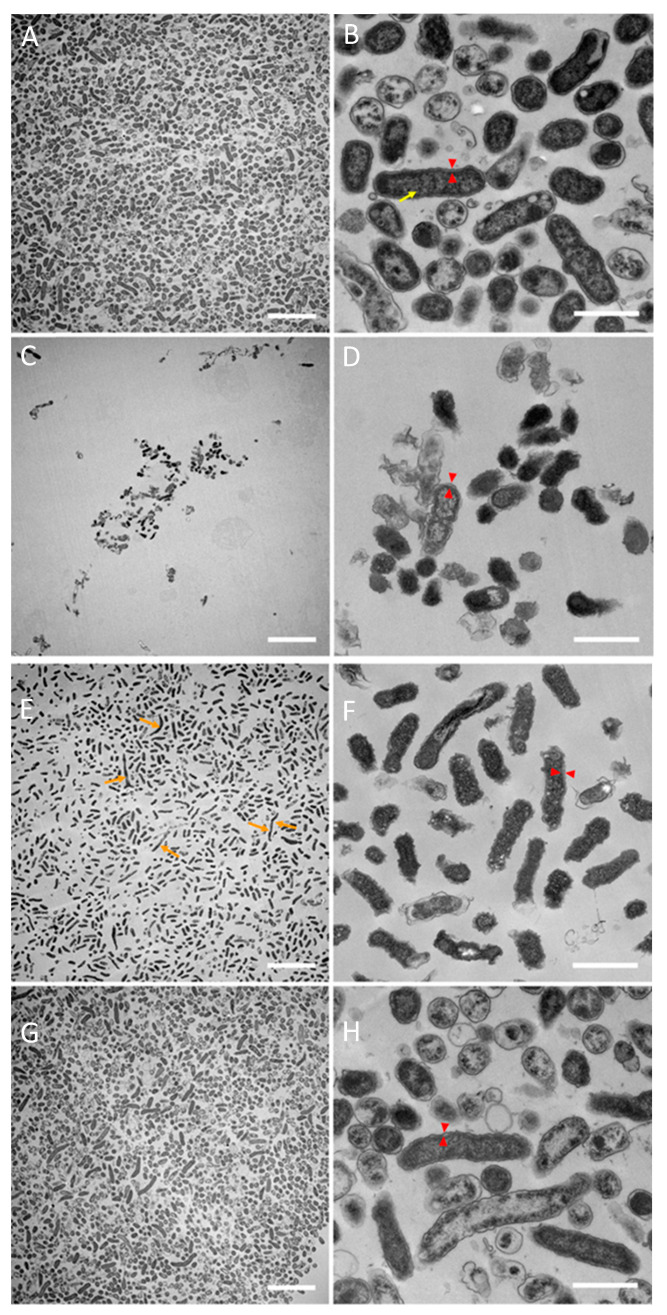
TEM of D7SS. The bacterial growth conditions and sample preparation are described in Figure 3 and include only the bacteria pelleted from cultures. The samples included D7SS in the mTSB control (panels **A**,**B**), in RPMI-1 (panels **C**,**D**), and RPMI-2 (panels **E**,**F**), and bacteria switched from RPMI-2 to mTSB (panels **G**,**H**). Cytoplasmic membranes, periplasmic space, and outer membranes are represented by red triangles, yellow arrows highlight the pale-staining area occupied by genomic DNA, and extra-long cells are highlighted by orange arrows. The scale bars on the left and right columns are 5 µm and 500 nm, respectively.

D7SS in the mTSB control showed intact cells that, on average, were longer than those of D7S-1 (Figure 6A,B). The cells had well-defined cytoplasmic membranes, periplasmic space, and outer membranes (red triangles). The intracellular content was characterized by a pale-staining area of DNA (yellow arrows) and numerous ribosomes. No fimbriae were found. D7SS in RPMI-1 showed very few cells and evidence of damage (Figure 6C,D). A few intact cells were found, characterized by poorly defined cell wall structures and irregular-shaped outer membranes (red triangles). Relatively few ribosomes were found in the cytoplasm. D7SS in RPMI-2 showed more cells than in RPMI-1 (Figure 6E,F). Occasional extra-long cells (orange arrows) were noted. The cell morphology was similar to that in RPMI-1, with the bacteria displaying irregular-shaped outer membranes. The morphology of the cells in RPMI-2 changed after reversion to the mTSB medium and resembled the cells in mTSB more closely (Figure 6G,H). Notably, the outline of the cells was smoother compared to the bacteria cultured in either RPMI-1 or RPMI-2.

## 4. Discussion

Bacteria cultured in complex media exhibit the typical bacterial lifecycle: a brief lag phase, followed by rapid proliferation during the exponential phase fueled by abundant nutrients. When resources diminish, the rapid growth slows, and bacteria transition into a stationary phase marked by the absence of growth. In a closed culture system, when nutrients are not replenished, bacteria will eventually enter the death phase where, in *Escherichia coli* K12 cultured in Luria Bertani medium, 99% of the viable cells will be lost within three days [10].

In their natural environment, periods of bacterial exponential growth are short as nutrients are typically limited and conditions are generally harsh [11]. Bacteria are often forced to remain in the stationary phase for an extended time [12] until conditions become more favorable. However, when conditions fail to improve, cells may progress into the death phase. The mechanisms underlying the transition from the stationary phase to the death phase are poorly understood [10]. One theory hypothesizes that whenever the bacterial load exceeds the support potential of the culture, cells begin to die as there are no sufficient resources to continue their metabolism and carry out the repair and maintenance functions. This death then releases cell contents, including amino acids from proteins, carbohydrates from the cell walls, lipids from cell membrane materials, and DNA, that can be utilized by the surviving siblings [10,13]. Another theory proposed that in a high-density culture, cells can sense that resources are becoming limited, and a proportion of cells will automatically go through altruistic suicide so that others can continue to grow utilizing the remaining nutrients and survive [10,11,13].

Despite the death of the majority of the population during the transition from the stationary to death phase, some proportions of cells may survive [11] and enter into dormancy instead. They are viable but non-replicating in this state and exhibit low metabolic rates with distinct gene expression profiles [14]. Metabolic activities slow down to a minimum to conserve energy and increase their likelihood of survival. This group of surviving cells makes up what is known as the seed bank, and it serves to ensure that at least some cells can be resuscitated when conditions improve in the future [14].

Dormancy, however, is an energetically expensive process [14]. Although energy production slows to the bare minimum, bacteria must still allocate resources to maintain cellular structures and machinery [14]. They still need to spend energy maintaining surveillance of their environment to detect the appropriate time to exit dormancy and resume normal metabolism [14]. Yet, this energy investment will almost guarantee their survival.

In this study, we attempted to generate an in vitro dormancy model for *A. actinomycetemcomitans*. Most in vitro bacterial studies have relied on the use of complex media, and this strategy has significant drawbacks in microbial research. First, the undefined nature of the complex medium components frequently impedes hypothesis testing. Additionally, complex media do not accurately simulate the nutrient constraints of the in vivo environment where bacteria often experience starvation. Therefore, we chose CDMs to test our hypothesis. The precise formulations of CDMs allow us to make specific modifications to develop different media with distinct growth support properties. RPMI-1640 was selected as the base of our CDMs because our previous study showed that this medium (i.e., RPMI-1 in this study) supported the viability of *A. actinomycetemcomitans* for 24 h, with no apparent growth, and induced a distinct gene expression profile [15]. RPMI-1 was further amended to derive RPMI-2 and RPMI-3, and these amendments were tailored to *A. actinomycetemcomitans* based on its genome analysis [16] and growth requirements of *Haemophilus influenzae* [17,18,19]. *H. influenzae* and *A. actinomycetemcomitans* are genetically related and may share similar growth requirements.

We first determined whether the media were nutrient-deficient or nutrient-limited using the conventional enriched medium mTSB as a control (Figure 1). The critical finding was the difference between the growth phenotypes of *A. actinomycetemcomitans* in RPMI-1 and RPMI-2. The exhaustion of essential growth factors cannot explain the difference. A key finding to the formulation of the media is the requirement of nucleotide synthesis via salvage pathways for *A actinomycetemcomitans*. RPMI-1640, amended with uracil and hypoxanthine, provided all necessary components for the growth of *A. actinomycetemcomitans*. The results suggested that RPMI-1 is an ND medium, while RPMI-2 is an NL medium. Our results for RPMI-1 differed from the observation by Sreenivasan et al. [20], which suggested that RPMI-1 could sustain *A. actinomycetemcomitans’* growth. However, in their study, the growth of *A. actinomycetemcomitans* was monitored for 24 h only, which did not allow for sufficient time to demonstrate the decline in viability over time.

The growth differences in D7S-1 between mTSB and RPMI-2 were statistically significantly different. However, the differences were less than those in D7SS in the same media. It appears that D7S-1 was more resistant to nutrient limitations, which was more evident in the experiment in Figure 2. *A. actinomycetemcomitans* exhibited distinct cellular morphology when cultured in mTSB, RPMI-1, and RPMI-2. While there were differences in the cellular morphology of D7S-1 and D7SS, and the growth conditions were not the same as those tested in growth studies, the results suggested that *A. actinomycetemcomitans* in RPMI-2 was largely intact, remained relatively healthy, and exhibited a morphology distinct from that in mTSB. Perhaps not surprisingly, *A. actinomycetemcomitans* grown in RPMI-1 was significantly stressed, with numerous cells showing extensive damage, and was not considered dormant as defined by our study. In RPMI-2, the morphology of *A. actinomycetemcomitans* showed characteristics that were somewhat between the morphology in mTSB and RPMI-1, and the phenotype was reversible if the bacteria were transferred back to complex mTSB medium; they became indistinguishable from those with no exposure to the nutrient-limited medium.

We noted the presence of thin fimbriae but never thick bundles of fimbriae in the smooth-colony D7SS in liquid cultures. The strain D7SS has a point mutation in the promoter region of the fimbria biogenesis operon [21]. The results suggested that the point mutation’s effect on fimbria expression is leaky, leading to the expression of thin fimbriae but not the thick bundles of fimbriae noted in the wildtype D7S-1. A similar observation that smooth-colony mutants expressed a lower abundance of and shorter fimbriae has been reported previously [22]. The role of the thin fimbriae in adherence and aggregation for *A. actinomycetemcomitans* remains to be elucidated.

Our lab routinely cultured *A. actinomycetemcomitans* in NE media for growth study and consistently observed a death phase typically after two days of incubation, when the bacteria were not recoverable. Therefore, the result of the sustained viability of D7S-1 in RPMI-2 was striking. Notably, the bacteria appeared to enter a phase of sustained viability starting from day 4, and the viability remained unchanged until the end of the experimentation. It is unknown how long the viability can last if we continue the experiment.

*A. actinomycetemcomitans* in RPMI-1 also exhibited a sustained but declining viability. This phenotype cannot be explained by the exhaustion of essential growth nutrients in the media because of the typical death phase encountered by *A. actinomycetemcomitans* grown in NE medium in 2–3 days. We hypothesize that the induction of *A. actinomycetemcomitans* dormancy requires both a specific formulation of the media and the presence of essential components to maintain dormancy.

There are several limitations and caveats to this study. The proportions of live and dead cells in EM studies were unknown. Future studies should include live/dead staining of both planktonic and biofilms in different culture conditions. The cellular morphology achieved by using EM was descriptive, and there is a need for quantitative analysis of the cells (e.g., cell size). Also, the pathogenesis of *A. actinomycetemcomitans* in periodontitis is complex. The simple model of this study does not simulate the complex in vivo growth condition, but will be useful for further investigation into the survival mechanism of *A. actinomycetemcomitans* in vivo.

Our study has not resolved when and how to distinguish dormancy and starvation and whether these conditions have overlapping phenotypes and mechanisms. These are the issues that require additional experiments for clarification. In the current study, we chose to avoid starvation by replenishing the media in the experiments to examine the cellular morphology by EM. We are currently investigating the genes involved in the phenotypes observed in this study by employing transcriptomics and Tn-seq. Once a list of candidate genes is identified, we will begin sorting out the genes involved in dormancy, starvation, or both. To the best of our knowledge, this is the first study to identify a growth phenotype of *A. actinomycetemcomitans* that resembles dormancy. Experiments are underway to investigate the essential genes and the transcriptomes of the putative *A. actinomycetemcomitans* dormancy. The information may provide insight into the pathogenesis of *A. actinomycetemcomitans* in periodontitis. Moreover, RPMI-1640 may be amended as a CDM for experiments that require specific information on each ingredient in the media. RPMI-1640 may also be tailored to other oral species based on genome analysis. We plan to test the CDMs developed in this study as growth media for in vitro polymicrobial models. These CDMs may be useful in investigating the complex bacteria-to-bacteria relationships in dental biofilms.

## 5. Conclusions

RPMI-1640 and its amendments are CDMs that can be used to test hypotheses related to the growth of *A. actinomycetemcomitans*. Specifically, we discovered *A. actinomycetemcomitans*’ dormancy-like phenotype when the bacteria were cultured in a nutrient-limited RPMI-1640 amended with bases. The *A. actinomycetemcomitans* dormancy-like phenotype is characterized by a reduced but stable viability without the death phase, in contrast to the bacteria grown in enriched laboratory media. The *A. actinomycetemcomitans* dormancy-like phenotype exhibits distinct cellular morphology examined by SEM and TEM. The dormancy-like phenotype is reversible upon culturing in enriched laboratory media. The in vitro dormancy model may be useful for investigating the mechanisms of the survival of *A. actinomycetemcomitans* in nutrient-limited conditions.

## Figures and Tables

**Table 1:** List of samples for EM.

Strain	Protocol	Timeline
D7S-1 and D7SS	Bacteria cultured in mTSB, RPMI-1, or RPMI-2 media with daily 1:1 dilution with fresh media.	Day 1–Day 4
D7S-1 and D7SS	Bacteria grown in RPMI-2 from Day 1–Day 4 above were transferred to mTSB with daily 1:1 dilution with the fresh medium.	Day 5–Day 7

## Data Availability

Data are contained within the article.

## Supplementary Materials

The following supporting information can be downloaded at: https://www.mdpi.com/article/10.3390/pathogens13050418/s1, Figure S1: Summary of protocols used to prepare *A. actinomycetemcomitans* samples for electron microscopy.
