# Supplementary material for: Dormancy-like Phenotype of Aggregatibacter actinomycetemcomitans: Survival during Famine
Source: Pathogens. 2024 May 16;13(5):418. doi: 10.3390/pathogens13050418 (PMC11124257; doi:10.3390/pathogens13050418)
Supplement: Supplementary file 1 [file pathogens-13-00418-s001.zip › pathogens-2922247-supplementary.pdf]

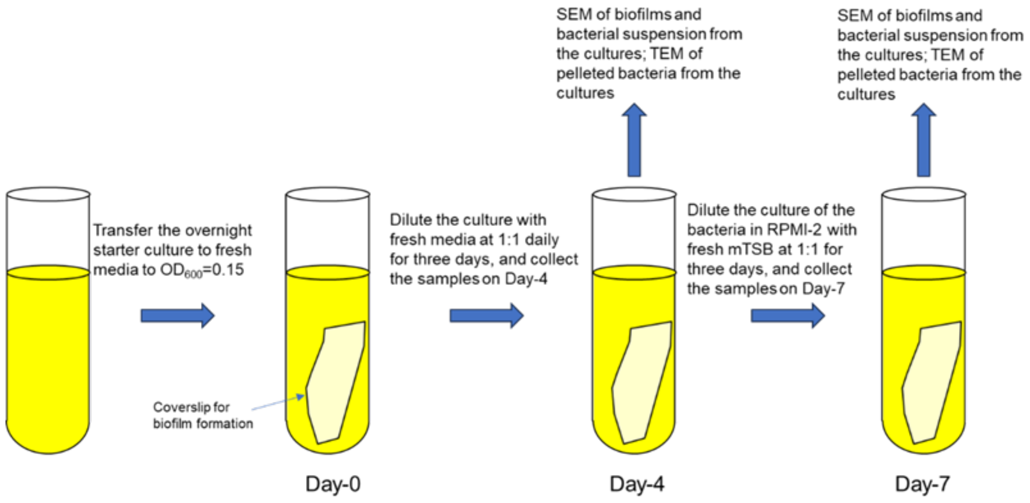

Supplementary Figure S1. Summary of protocols used to prepare *A. actinomycetemcomitans* samples for electron microscopy.
